# Virtual joint school prior to hip and knee arthroplasty: patient feedback and carbon footprint savings

**DOI:** 10.1308/rcsann.2025.0080

**Published:** 2026-01-20

**Authors:** D Pinto, M Clarke, M Ganapathi

**Affiliations:** ^1^ Hywel Dda University Health Board, UK; ^2^ University Hospitals of North Midlands NHS Trust, UK; ^3^ Betsi Cadwaladr University Health Board, UK

**Keywords:** Arthroplasty, Patient education, Carbon footprint

## Abstract

**Introduction:**

Patient education programmes prior to hip and knee arthroplasty reduce anxiety and create realistic expectations. We describe a ‘virtual joint school’ (VJS) model and analyse patient feedback and environmental impact.

**Methods:**

Eligible patients first viewed online educational videos, and then attended an interactive virtual session during which knowledge was reinforced. Each session was attended by eight to ten patients along with a relative or friend, and was hosted by a multidisciplinary team consisting of nurses, physiotherapists, occupational therapists and a former patient who provided personal insight. Feedback was obtained prospectively using an electronic questionnaire, and travel savings were calculated using Python software.

**Results:**

From July 2022 to February 2023, 267 patients attended the VJS; of whom 117 (44%) responded to the questionnaire. Among them, 87% found the pre-learning videos helpful and comprehensible, 92% felt their concerns were adequately addressed, 96% felt they had sufficient opportunity to ask questions and 96% were happy with the level of confidentiality involved. Although 83% felt they received sufficient support from the health board to access the virtual session, 63% also took support from family/friends to attend it. Only 15% felt they would have preferred a face-to-face format. By having ‘virtual’ sessions, each patient saved, on average, 38 miles and 62min of travel (10,070 miles and 274h saved for 267 patients). Each VJS session produced 0.32kg of CO_2_ compared with 110kg of CO_2_ per face-to-face session.

**Conclusions:**

Virtual joint schools are acceptable to patients and reduce the carbon footprint of healthcare. We recommend their implementation at other arthroplasty centres.

## Introduction

Surgery schools prior to hip and knee arthroplasty have been shown to have numerous benefits. They give patients a better understanding of the planned surgery, thus creating realistic expectations and reducing anxiety.^1,2^ They have been shown to be associated with improved postoperative pain control, reduced length of stay in hospital and a decreased rate of readmissions.^1,3–5^ Modern joint schools also incorporate ‘prehabilitation’, which involves optimising physical fitness, nutrition and emotional wellbeing prior to surgery, and has been shown to further improve surgical outcomes.^6,7^ The Royal College of Surgeons of England, in a joint statement issued in June 2021, recommended that all patients being considered for major elective surgical interventions should be invited to attend group ‘surgery schools’.^8^

Prior to the COVID-19 pandemic, we had been running group ‘joint schools’ before total knee replacements (TKR) and total hip replacements (THR) at our institute for a number of years. These were run in a face-to-face format and were generally well received by patients. However, there were several disadvantages to this format. The face-to-face joint schools are time-consuming, not only for patients, but also for the multidisciplinary team (MDT) running them. They require patients with generally poor mobility to travel to the hospital, often over significant distances given our health board’s wide catchment area. Travelling to the hospital also often required patients to be accompanied by relatives or friends, who in turn needed to take time off work. The joint schools also provide a large volume of information in a short period, which can be difficult for patients to process in a single sitting.

With the advent of the COVID-19 pandemic, these face-to-face joint schools were no longer feasible. Use of a virtual model was thus envisaged, so that the joint schools could continue while maintaining social distancing. The virtual format was also in keeping with the recent trend for adoption of telemedicine modalities to mitigate the environmental impact of health services.

Since 2021, we have been running the virtual joint school (VJS) twice weekly, for patients awaiting hip and knee arthroplasty. The aim of this study was to analyse patient acceptability of the VJS model, and to determine its impact on environmental sustainability.

## Methods

### VJS model

Our VJS consisted of two parts. Patients due to undergo elective hip or knee replacements were first invited to view a series of educational videos, made available online through YouTube. These videos were created by our arthroplasty MDT, consisting of arthroplasty coordinator, lead arthroplasty nurse, anaesthetists, physiotherapists, occupational therapists and dieticians. Two sets of videos were created, providing information specific to hip and knee arthroplasty, respectively. A list of the included videos for both knee and kip arthroplasty is provided in Table 1.

**Table 1:** Videos for knee and hip arthroplasty

Knee replacement joint school videos	
1	Lifestyle programme to lose weight before joint replacement
2	Getting match fit – how to optimise your health for major surgery
3	Knee replacement – your journey explained
4	To sleep or not to sleep – all about your anaesthesia
5	Knee replacement exercises
6	Mobilising with crutches
7	Occupational therapy – knee precautions
8	The paperwork
Hip replacement joint school videos	
1	Lifestyle programme to lose weight before joint replacement
2	Getting match fit – how to optimise your health for major surgery
3	Hip replacement – your journey explained
4	To sleep or not to sleep – all about your anaesthesia
5	Hip arthroplasty exercise guide
6	Mobilising with crutches
7	Occupational therapy – hip precautions
8	Occupational therapy – using aids
9	How to use dressing aids
10	How to get on and off the bed following your hip replacement
11	Getting in and out of a car following hip replacement surgery
12	The paperwork

Following this, patients were invited to attend an interactive virtual session that was run using Microsoft Teams software. During this session, knowledge from the videos was reinforced, further information was provided and patients were given the opportunity to ask questions. Each virtual session was typically attended by eight to ten patients, each being accompanied by family members/friends if they so desired. Sessions were hosted by the same MDT members. In addition, a patient who had previously undergone hip or knee arthroplasty was also invited to speak at the session, to provide insight from the patient perspective. Each session was, on average, 1h in duration. After each talk by the MDT members, patients were given the opportunity to voice their concerns and ask questions, and time was also reserved at the end of the session for any lingering doubts. If there was any question that the MDT members could not answer, the question was ‘parked’, the answer was sought from the relevant professional, and the response emailed to all those that had attended that session of the VJS.

### Assessing the VJS model

A short electronic questionnaire was devised to evaluate the VJS model in terms of ease of access, usefulness and overall experience. The questionnaire was administered prospectively to patients attending the joint school over the course of 10 months. When filling the questionnaires, the participants were notified about the purpose of data collection and the potential for future use of the data for research purposes. The questionnaires were anonymised, and no patient-identifiable information was obtained, hence formal ethics committee approval was not required. A copy of the patient questionnaire is provided in Appendix 1 (available online).

To study the environmental impact of the VJS, we collected addresses of all patients attending the joint school in an anonymised manner. Using this information, we were able to calculate how much distance each individual patient would have needed to travel to and from the hospital, had the joint school been conducted face-to-face. Similarly, we could calculate how much travel time each patient had saved. These calculations were done using Python software. By inputting the patient address and hospital address postcodes, as well as the dates and times that the meetings were held, the software utilised Google maps to calculate the travel times and distances saved. The estimates were made based on real-time maps, thus taking account of actual routes that patients would have taken (rather than as the crow flies), and even factoring for traffic conditions on the days that the meetings were conducted.

### Data analysis

The questionnaires were distributed in Microsoft Forms format. Information from them was collated into Excel sheets for analysis.

## Results

From July 2022 to February 2023, 267 patients attended the VJS, and of these, 117 (44%) responded to the questionnaire. Attendance rate at the interactive virtual session of the VJS was 77% of those invited (71% of female patients and 81% of male patients). The average age of patients attending the VJS was 66 years among both women and men, with a range of 30–88 years for women and 48–81 years for men. In contrast, the average age of those who were invited but did not attend the VJS was 74 years (72 years for women and 78 years for men), with a range of 56–89 years for women and 71–88 years for men. For the 290 joint replacements (161 THRs and 129 TKRs) performed over the period of our study, 72% of these patients (total 208: 110 undergoing THR and 98 undergoing TKR) attended the VJS prior to their surgery.

**Figure 1:**
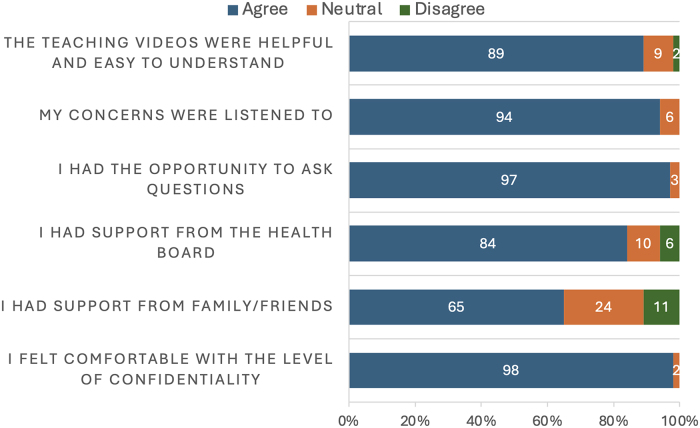
Patient feedback for the virtual joint school

Of the respondents, 87% found the pre-learning videos helpful and easy to understand, 92% felt that their concerns were heard and addressed, and 96% felt they had sufficient opportunity to ask questions. From a technical perspective, 83% of respondents felt that they received sufficient support from the health board to access the virtual teaching sessions, 63% were able to get support from family/friends to attend the virtual session and 96% were happy with the level of confidentiality involved in the programme. Only 15% of respondents felt that they would have preferred a face-to-face format for the joint school. Patient feedback to the VJS is provided schematically in Figure 1.

Participants were asked to quantify how prepared they felt for their surgical journey, on a scale of 1 to 10 (with 10 being the best); and the responses improved from a median of 7 before attending the joint school, to 9 after attending it. Some examples of the comments made by patients when providing feedback about the VJS are provided in Table 2.

**Table 2:** Examples of patient comments about the virtual joint school

Thought that the experience was great knowing that others were in the same situation, and I was not the only one.
Different people in the class had different questions that I had not thought about. So, between the class a wide variety of questions were put forward, making it a very informative session.
I felt it gave me a real connection with the forthcoming procedure.
It was reassuring to hear from other patients who had similar concerns, and from one person who was very positive about her previous experience of surgery.
This is the future when having appointments where actual face-to-face is not necessary. Saving both the client and hospital staff time.

By having the sessions conducted virtually rather than face-to-face, each patient saved, on average, 38 miles and 62min of travel. In other words, for the 267 patients that attended the VJS over this period (and responded to our questionnaires), there were total savings of 10,070 miles and 274h of travel. Using online carbon footprint calculators, we determined that each session of the VJS (ten patients attending) would produce the carbon dioxide equivalent (CO_2_e) of just 0.32kg (CarbonFreeConf™; https://www.carbonfootprint.com/calculator.aspx), compared with 110kg CO_2_e, per session if conducted in a face-to-face format (Carbon Footprint Ltd; https://www.carbonfootprint.com/calculator.aspx).

## Discussion

Primary total hip and knee replacements are some of the most common elective orthopaedic procedures carried out in the UK. Considering the current annual rates at which these procedures are performed, and allowing for projected changes in population demographics, it is estimated that there will be more than 200,000 THRs and TKRs performed in the UK in 2035.^9^ This places considerable demands on the National Health Service (NHS), and we need to incorporate newer technologies to keep up with this demand while ensuring that patient outcomes are optimised.

Although extremely effective in improving lifestyle and productivity in patients with crippling arthritis, these procedures also have an undeniably negative environmental impact.^10,11^ Travel associated with healthcare is itself a major source of greenhouse gas emissions, with patient travel contributing to 5% of the NHS’ ‘carbon footprint plus’ (emissions produced by the NHS directly plus emissions it can influence).^12^ In January 2020, the For Greener NHS campaign was launched, with the goal of achieving zero emissions over the entire scope of the NHS carbon footprint by 2045.^12^ Telemedicine modalities can be used to reduce the carbon footprint of healthcare, and an exciting avenue for their use is in the preoperative education of patients undergoing TKRs and THRs.^13^

We implemented the VJS model to provide preoperative education to patients undergoing TKRs and THRs in an interactive but socially distanced manner, while simultaneously mitigating the impact of this service on the environment. The results of this study show that we have been successful in achieving these goals.

We used a two-phase approach to patient education. The first phase, using YouTube videos to provide preoperative education regarding hip and knee arthroplasty, has been described in the past, and has been shown to have beneficial effects in reducing anxiety.^14^ This was borne out in our study, with 89% of respondents agreeing that the online teaching videos were helpful. The purpose of providing information through videos, rather than through printed literature, was to make it more interesting and appealing, to maximise the amount of information provided and give easily visualised demonstrations.

The second phase consisted of the interactive group sessions. In these sessions, patients could interact with others in similar positions to themselves, which helped foster a sense of solidarity and hope. They heard from patients who had previously been through a similar treatment journey and could benefit from their insight and experience. The sessions were designed to be as interactive as possible, and all patients were encouraged to voice their concerns and anxieties, with more than 90% of respondents agreeing that they were satisfied in this respect. Furthermore, the MDT team provided advice on optimising physical and mental health in preparation for major surgery and encouraged patients to set personal goals to achieve this.

Running the joint school in a virtual format did present some challenges. MDT staff needed to be trained on the technical aspects of running the VJS, and in turn reached out to individual patients to provide technical support and guidance to those unfamiliar with the use of these virtual platforms; 84% of respondents agreed that this was adequate. A number of participants also had support from family and friends in this regard. Nevertheless, it was recognised that a proportion of patients were unable to attend the virtual sessions despite being offered support. It is telling that the average age of those that did not attend the VJS was higher than the average age of those that did attend (74 years vs 66 years), which may be because the older patients in our cohort were less proficient in the use of computers or smartphones. Indeed, 15% of those that did attend the virtual sessions admitted that they would have preferred to have the sessions face-to-face. As a result, we continue to run at least one face-to-face joint school session per month, targeting those that are unable to attend the VJS.

The concept of using digital technologies to provide preoperative education in hip and knee arthroplasty is not new. Anderson *et al* developed a prototype ‘virtual knee school’ that was found to be acceptable to most participants.^15^ Gray *et al* demonstrated an improvement in patient-reported outcome measures (PROMs) and a reduction in length of hospital stay among patients who utilised a ‘digital joint school’ education programme, compared with those given standard written and verbal preoperative information.^16^ Although the provision of patient education resources in the form of videos, PDF documents, forms and questionnaires is common across various joint schools, a unique feature of our VJS model is the interactive virtual sessions, which gave patients the opportunity to interact with others in similar situations to themselves, and empowered them to ask questions. Furthermore, in addition to demonstrating patient satisfaction with the VJS format, ours is the first study to demonstrate the beneficial effects of this virtual format on reducing the carbon footprint of healthcare.

### Study limitations

This study had some limitations. We had a limited response rate to the electronic survey, which might reflect the lack of familiarity with digital platforms that is an issue for patients in this cohort. We made several assumptions when calculating the estimated carbon footprint savings. The online calculator provided an estimate of carbon emissions based on the assumption of an average sized car, using an unknown fuel. These might be slightly different in real terms, depending on the actual vehicles used, their ages and so on. Besides, some patients may have utilised public transport to make their way to and from the hospital. Nevertheless, the stark difference in the CO_2_ output suggests that, even if these calculations are not completely accurate, the virtual model is certainly better towards achieving the NHS goal of sustainability.

It is important to note that the CO_2_ offset by running virtual meetings is heavily influenced by the geographical spread of the population being served. Hospitals such as ours that cater to a wider geographic area have the most to gain by running virtual rather than face-to-face clinics.

Several unintended but pleasant side effects of the VJS have been discovered since its inception. Having the school run virtually has allowed patients’ relatives to be much more involved in their treatment journey. It has been possible to begin patient education and prehabilitation very early in their treatment journey – patients can be directed to access the online resources right at the time of initial listing for surgery. Through liaison with general practitioner (GP) practices within the health board, this can even be extended to the point of contact with their GPs.

Finally, the success of the VJSs has opened several further avenues of improvement. We plan to initiate virtual ‘waiting well’ clinics, to provide pain management and health optimisation strategies for patients awaiting surgery. The digital transformation can also be extended to digital consenting, digital collection of PROMs and digital follow-up. All of these measures align with the long-term plan of bringing digitally enabled care to the mainstream within the NHS.^17^

## Conclusion

This VJS model was devised to provide pre-arthroplasty education in a socially distanced and environmentally friendly manner. This study has presented patient feedback and sustainability data that show this model is valid and achieves its stated objectives. We advocate the adoption of similar VJSs at other arthroplasty centres as well.
